# Simulation of a Multiband Stacked Antiparallel Solar Cell with over 70% Efficiency

**DOI:** 10.3390/ma18245625

**Published:** 2025-12-15

**Authors:** Rehab Ramadan, Kin Man Yu, Nair López Martínez

**Affiliations:** 1 Universidad Autónoma de Madrid, C/Francisco Tomás y Valiente 7, 28048 Madrid, Spain; nair.lopez@uam.es; 2 Department of Physics, Faculty of Science, Minia University, Minia 61519, Egypt; 3 Department of Physics, National Sun Yat-sen University, Kaohsiung 80424, Taiwan; kinmanyu@cityu.edu.hk; 4 Departamento de Óptica y Fotónica de Superficies y Materiales, Instituto de Óptica—CSIC, C/Serrano 121, 28006 Madrid, Spain

**Keywords:** highly mismatched materials, three active energy band transitions, stacked-antiparallel junctions, reverse tunneling current, multiband solar cells

## Abstract

**Highlights:**

**What are the main findings?**

- New multiband cell removes tunnel junctions with stacked anti-parallel junctions.
- Uses GaAsN’s three-band structure for bidirectional carrier generation.
- Blocking layers stop contact recombination while preserving triple absorption.

**What are the implications of the main findings?**

- Rivals six-junction cell performance with a far simpler fabrication process.
- Establishes highly mismatched alloys as a platform for next-gen photovoltaics.
- Record 70% theoretical efficiency under concentration, per SCAPS-1D simulations.

**Abstract:**

Multiband solar cells offer a promising route to surpass the Shockley-Queisser limit by harnessing sub-bandgap photons through three active energy band transitions. However, realizing their full potential requires overcoming key challenges in material design and device architecture. Here, we propose a novel multiband stacked anti-parallel junction solar cell structure based on highly mismatched alloys (HMAs), in particular dilute GaAsN with ~1–4% N. An **anti-parallel junction** consists of two semiconductor junctions connected with opposite polarity, enabling bidirectional current control. The structures of the proposed devices are based on dilute GaAsN with anti-parallel junctions, which allow the elimination of tunneling junctions—a critical yet complex component in conventional multijunction solar cells. Semiconductors with three active energy bands have demonstrated the unique properties of carrier transport through the stacked anti-parallel junctions via tunnel currents. By leveraging highly mismatched alloys with tailored electronic properties, our design enables bidirectional carrier generation through forward- and reverse-biased diodes in series, significantly enhancing photocurrent extraction. Through detailed SCAPS-1D simulations, we demonstrate that strategically placed blocking layers prevent carrier recombination at contacts while preserving the three regions of photon absorption in a single multiband semiconductor p/n junction. Remarkably, our optimized five-stacked anti-parallel junctions structure achieves a maximum theoretical conversion efficiency of 70% under 100 suns illumination, rivaling the performance of state-of-the-art six-junctions III-V solar cells—but without the fabrication complexity of multijunction solar cells associated with tunnel junctions. This work establishes that highly mismatched alloys are a viable platform for high efficiency solar cells with simplified structures.

## 1. Introduction

Efficient solar energy conversion is fundamentally limited by the broad solar spectrum (0.5–4 eV). While single-bandgap photovoltaics are constrained by the Shockley-Queisser limit [1], III-V multijunction solar cells (MJSCs) achieve record efficiencies (>47% under concentration [2,3,4]. However, high efficiency MJSCs based on thin films or nanostructured materials require complex epitaxial growth and efficient tunnel junctions, which hinder their scalability [5,6]. The novel concept of a single junction solar cell with materials with multiple energy bands (multiband solar cells) offer an alternative [7,8,9,10], yet carrier extraction in this design remains challenging. The development of multiband solar cells has been pursued through methods such as the theoretical modeling of quantum dot intermediate band cells [11], the fabrication of single junctions with intermediate band materials [12], and tandem cell designs. These approaches, however, typically involve complex fabrication and rely on critical tunneling junctions [13,14,15,16].

**Figure 1:**
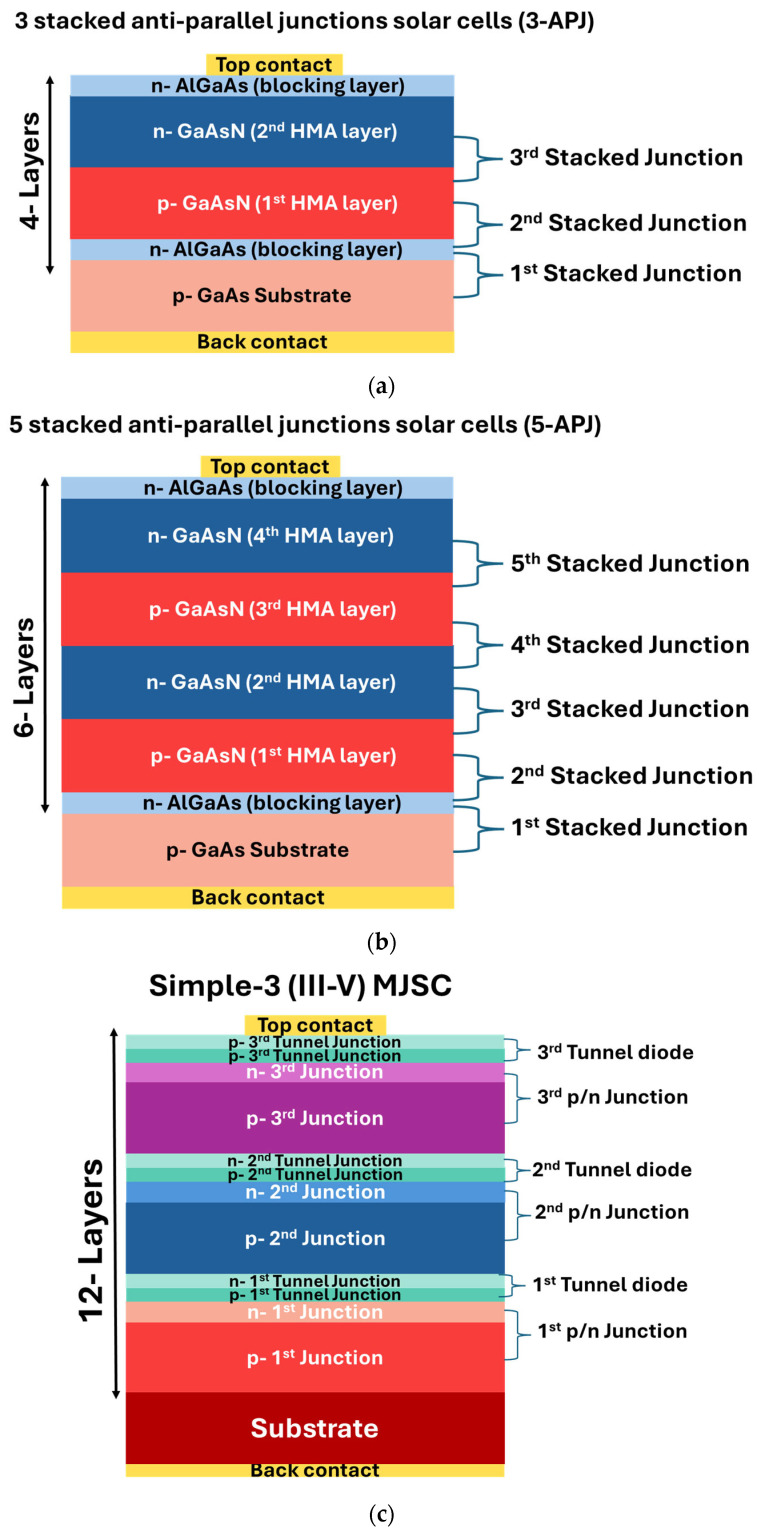
Schematic representation of the difference between the novel (**a**) 3 stacked anti-parallel junctions (3-APJ) and (**b**) 5 stacked anti-parallel junctions structures (5-APJ) and (**c**) a simple 3- (III-V) MJSCs. The number of layers in each structure is indicated in the figures.

Here, we present a new multiband stacked anti-parallel junctions solar cell architecture using highly mismatched alloys (HMAs, such as GaAsN). In GaAsN HMAs, when a small fraction (a few %) of As in GaAs is replaced by more electronegative, equivalent N atoms, anticrossing interactions of the N localized states and the host GaAs conduction band (CB) states result in splitting of the CB. As a consequence, the alloy has three optically active energy bands, namely the original valence band (E_V_), a lower (E_−_(k)) and a higher (E_+_(k)) conduction band. This multiband structure facilitates more carrier generation when the HMA layers are properly confined by wide-bandgap blocking layers (e.g., AlGaAs for GaAsN systems) [17,18,19]. Crucially, this new multiband stacked anti-parallel junctions solar cells enable bidirectional carrier generation—under both forward and reverse bias—via a current tunneling mechanism across the three active energy bands, and thus eliminating the need for tunnel junctions, which are crucial in conventional multijunction solar cells. Figure 1 shows schematic diagrams representing our proposed cell structures. The structure with 3 anti-parallel junctions (3-APJ) (Figure 1a) is formed by a total of four grown layers in addition to the substrate and the back and front ohmic contacts. On a p-GaAs substrate a blocking layer of n-AlGaAs is grown, followed by a layer of p and then a n GaAsN layers, and then finally covered with another n-AlGaAs blocking layer. Here, anti-parallel junctions refer to the n-p/p-n/n-p junctions (from the top n-GaAsN layer) with opposite polarity as shown in Figure 1a. A 5 stacked anti-parallel junctions structure (5-APJ), n-p/p-n/n-p/p-n/n-p, was formed by a total of six grown layers, the substrate and the front and back contacts are shown in Figure 1b. In this case, a second p-n GaAsN junction pair was grown on top of the first GaAsN p-n junction in Figure 1a. The full structure is formed by 5 stacked anti-parallel junctions (5-APJ).

Finally, to provide a direct comparison between the MJSC structure and our proposed structures, a schematic diagram showing a conventional 3-junction solar cell is shown in Figure 1c. Note that this MJSC structure contains 12 layers in addition to the substrate (plus the two ohmic contacts) with different materials, and these layers have to be lattice matched, thus making the fabrication process very complex and challenging.

Through SCAPS-1D simulations (SCAPS-3311), we optimize carrier generation/recombination rates in GaAsN layers and compare the performance of the 3-APJ and 5-APJ designs to conventional III-V solar cells with record power conversion efficiencies [4,10,20,21,22]. By adjusting the thickness of both the HMAs and the blocking-layers, N% in GaAsN, doping level, and metal contact work functions, we achieve efficiencies approaching theoretical limits for three-bands systems (∼63% under the maximum concentrated light [10]), and rivaling conventional III-V tandems structures [23,24,25,26].

## 2. Materials and Device Structure

Simulation details, including the simulation method [27], materials parameters considered to design the numerical model [8,18,28,29,30,31,32,33,34,35] and carrier transition mechanisms, are provided in the Supplementary Materials section.

**Figure 2:**
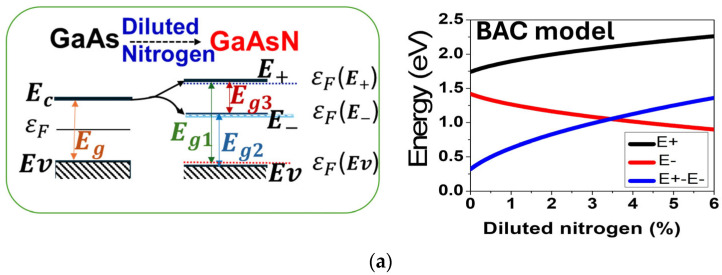

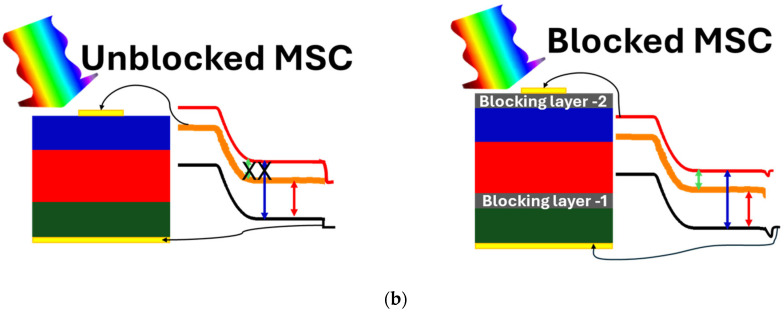
(**a**) A schematic illustration of the conduction band splitting induced by the incorporation of a diluted amount of nitrogen into the GaAs layer (**left panel**), alongside the different band positions as a function of N content in GaAsN computed by the band anticrossing (BAC) model (**right panel**). (**b**) Effect of blocking layers on carrier transport in a multiband GaAsN single p/n junction under illumination.

Highly mismatched alloys (HMAs) such as dilute nitrides (e.g., GaAsN) exhibit a split conduction band (E_±_(k)) as explained by the band anticrossing (BAC) model, enabling three optically active energy bands [8,12] when the HMA layers are sandwiched by blocking layers, as illustrated in Figure 2a,b. The electronic band structures of GaAsN with different N contents were computed based on the BAC model [18], using a MATLAB tool (MATLAB R2024b). Equation (1) shows the BAC model.

(1) $$E\pm =\frac{1}{2}(E_{N}+E_{M}\pm \sqrt{{(E_{N}-E_{M}}^{2})+4{C^{2}}_{MN}}X)$$

where E_N_ represents the energy of the localized nitrogen level (0.2%), E_g_ signifies the energy gap of GaAs matrix, and C_MN_, the coupling constant, is assigned a value of 1.5 eV. Additionally, X corresponds to the nitrogen concentration within the alloy (range from 0 to 6%).

As shown schematically in Figure 2a, the conduction band of GaAs splits into two subbands (E_−_ and E_+_) when a small fraction of As is replaced with N. Various electronic transition energies in a GaAsN layer as a function of N content are shown in the right panel of Figure 2a. Unlike conventional p/n junctions, blocking layers (e.g., AlGaAs) are needed to isolate the midband (E_−_(k)), forcing carrier transport through the new conduction band (E_+_(k)). The aluminum composition in the AlGaAs alloy was carefully optimized to achieve a layer with an energy bandgap of 1.98 eV, closely matching the E_+_–E_v_ bandgap of GaAsN (1.86 eV) at 1% nitrogen concentration, and also ensuring effective band alignment. The N% in GaAsN alloys is the same in all the GaAsN layers. Figure 2b presents the structures and energy band diagrams of a single p/n GaAsN junction, both with and without blocking layers, showing essential differences in the electronic transitions and conduction mechanisms in these structures. The inclusion of blocking layers disrupts the coupling between the midband E_−_ and E_+_ states, thereby inhibiting carrier transport from E_−_ to the device contacts. As a result, transitions in three optically active energy bands become possible, enhancing carrier generation efficiency.

**Figure 3:**
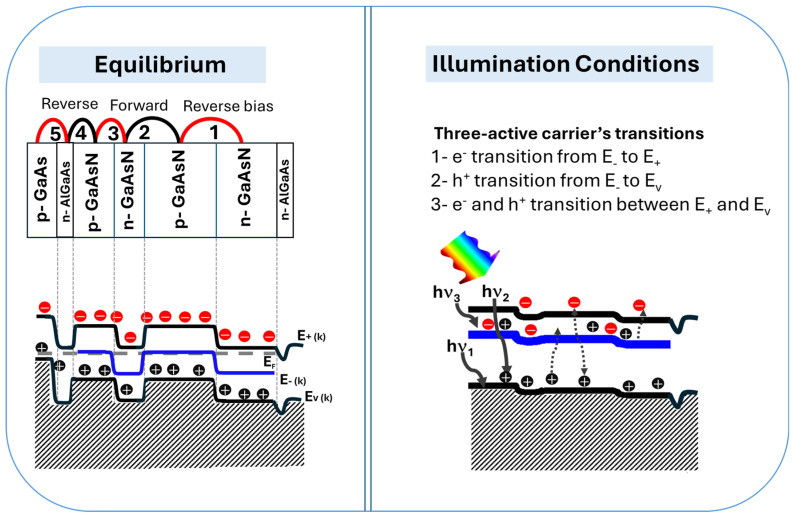
Energy band diagrams at equilibrium and under illumination conditions for the 5-APJ focusing on the 4 layers of GaAsN and blocked with AlGaAs blocking layers, highlighting mechanisms of light absorption, carrier transitions, and tunneling currents.

Figure 3 shows the energy band diagrams of the 5 APJ structure with the GaAs substrate, AlGaAs blocking layers and GaAsN layers. The 5 junctions are labeled 1–5 (from the top to the bottom) in the figure. The top 3 p/n junctions of the 5 total junctions at equilibrium and under illumination are shown. This structure significantly improves carrier generation due to the enhanced absorption of photons in different spectral range as well as suppresses recombination rates. Among the three junctions formed by GaAsN layers, the top GaAsN p/n junction exhibits the most favorable alignment. More specifically, under illumination the forward-biased junction shifts upward while the reverse-biased junction (junction 2) shifts downward, leading to an optimal band alignment. This configuration facilitates efficient carrier transitions across the three optically active energy bands. Consequently, the narrow energy tail observed between the top GaAsN layer and the adjacent layer in equilibrium is eliminated, mitigating potential recombination pathways across the band structure. Due to the series connection of the p/n junctions, a bidirectional photocurrent is observed, even without dedicated tunneling junctions. This includes a reverse component—referred to as a tunneling current—resulting from carrier tunneling transitions between the three energy bands.

The performance of these proposed new structures is evaluated by simulation by varying the materials parameters, including layer thickness, N% in GaAsN layers, carrier concentrations, number of junctions and work function of the metal contacts. Each parameter played a crucial role in the optoelectronic performance of the simulated multiband stacked anti-parallel junctions solar cells. The GaAsN alloy’s band gap and the E_−_(k) band position were selected based on the BAC model, determined by the nitrogen concentration in the alloy [18,35]. Materials parameters achieving optimized photovoltaic behavior and conversion efficiency in the simulations are summarized in Table 1.

## 3. Results

Leveraging this innovative solar cell architecture, we introduce a multiband solar cell featuring a multiple stacked anti-parallel junctions solar cell that operates without tunnel junctions. Two structures were examined via SCAPS 1-D simulations: one formed by 3 stacked anti-parallel junctions (3-APJ) (Figure 1a), and the other utilizing 5 stacked anti-parallel junctions (5-APJ) (Figure 1b). In both designs, GaAsN HMA p/n layers are strategically sandwiched between two AlGaAs blocking layers, with the entire structure grown on a p-GaAs substrate. By eliminating tunnel junctions, this approach simplifies fabrication while maintaining efficient photocarrier extraction. After systematic optimization of layer thickness, doping concentrations, and the number of stacked junctions, under 100-sun illumination, the 3-APJ and 5-APJ structures achieved a power conversion efficiency of 65% and 70%, respectively. These results establish a clear pathway toward the development of high-efficiency multiband solar cells based on anti-parallel junctions stacks. One of the crucial parameters determining the device performance is the positions of bands which can be adjusted by tuning the N content in the HMA as predicted by the BAC model, as illustrated in Figure 2. However, the addition of diluted nitrogen in GaAs to form GaAsN HMAs also introduces defects that create localized states within the bandgap due to non-uniform nitrogen distribution, which may act as trapping centers for charge carriers. These states may give rise to Shockley-Read-Hall (SRH) recombination, reducing carrier lifetimes and degrading optoelectronic efficiency [36,37].

### 3.1. Photovoltaic Performance

In order to obtain theoretical simulation results that are experimentally achievable, fabrication processes for the simulated devices are considered. HMAs are typically grown using epitaxial growth techniques such as molecular beam epitaxy or metal–organic vapor phase epitaxy, which ensure high precision in composition, thickness, doping concentration and good epitaxial quality, thereby minimizing the density of defects. The value of 10^18^ cm^−3^ used as our high-defect scenario was selected as a simulation parameter based on literature reports for similar highly mismatched III-N-V alloys (e.g., GaInNAs), where deep-level transient spectroscopy (DLTS) and photoluminescence studies have estimated total defect densities in the range of 10^14^ to 10^18^ cm^−3^ [36,37]. We adopted the upper limit as a conservative, worst-case estimate to model the potential impact of non-radiative recombination, acknowledging that high-quality MBE-grown material may have lower values.

**Figure 4:**
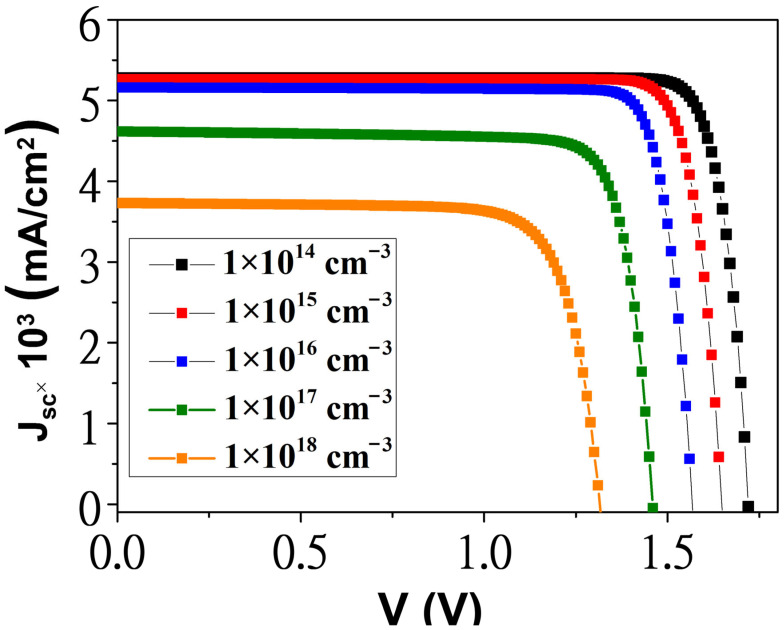
Photovoltaic behaviors of the 5-APJ at different defect densities in the GaAsN layers under 100 suns.

Figure 4 shows effects of increasing defect densities in GaAsN layers, ranging from 10^14^ to 10^18^ cm^−3^ on current-voltage characteristics for a 5-APJ structure under 100 sun illumination. Simulation results from Figure 4 summarized in Table 2 demonstrate that achieving around 80% conversion efficiency is feasible with a well-epitaxially grown stacked anti-parallel junctions structure.

**Figure 5:**
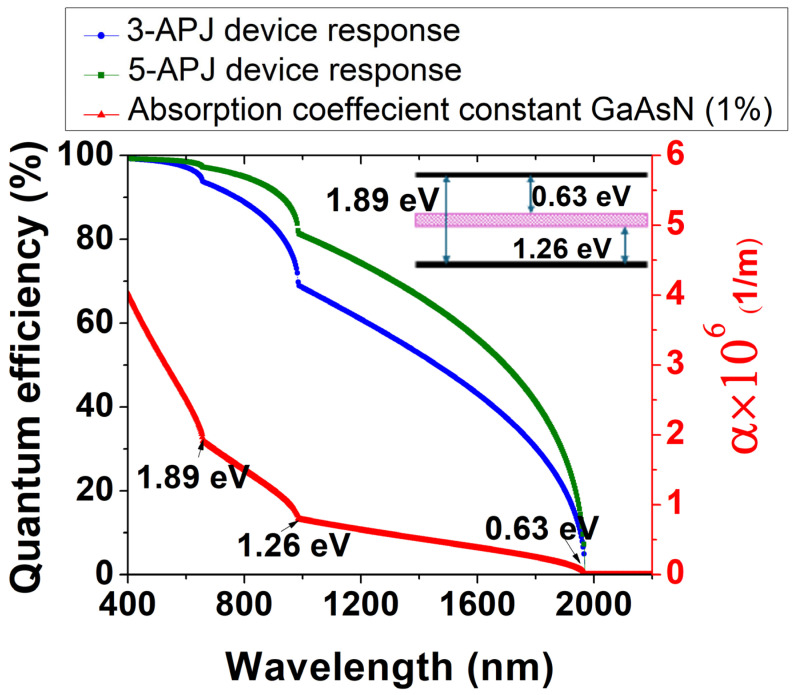
Spectral dependence of external quantum efficiency for 3-APJ and 5-APJ and absorption coefficient of GaAsN (1% N). The inset shows the corresponding band structure, highlighting the three optically active energy bands.

External quantum efficiency (EQE) is a pivotal parameter for solar cells, encapsulating the intricate interplay of absorption and recombination processes. Figure 5 presents a comparison between the EQE of the 3-APJ and 5-APJ structures. The absorption coefficient for GaAsN HMA with three optically active energy bands (gaps at 0.63, 1.26 and 1.89 eV) corresponding to a N content of 1.0% is also presented in the same figure. The remarkable photoresponse in the NIR region (>1000 nm) is attributed to the presence of the midband (E_−_(k) at 1.26 eV from the valence band) derived from the incorporation of diluted nitrogen in GaAs. The E_−_(k) band plays a crucial role in absorbing lower energy photons, thereby significantly boosting carrier generation rates. For this material, photons with energy as low as 0.63 eV (or a l of 1960 nm, corresponding to the energy bandgap between E_−_(k) and E_+_(k)) can be efficiently absorbed. EQE performance as a function of wavelength mirrors the absorption behavior in GaAsN layers with a 1% nitrogen concentration, highlighting the intricate relationship between material composition and photovoltaic efficiency.

A key to this superior solar cell performance is the suppression of non-radiative recombination in the HMA layers, which can be achieved through strategic band alignment by adjusting both GaAsN and AlGaAs layers composition.

### 3.2. Energy Band Engineering and Carrier Dynamics

**Figure 6:**
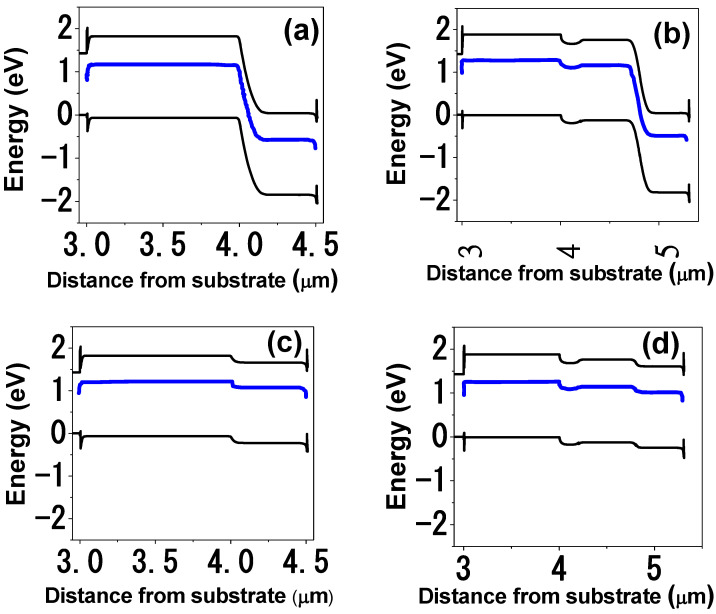
Energy band diagrams of (**a**) the 3- and (**b**) 5-APJ structures in dark without illumination and (**c**,**d**) under illumination. Blue lines indicate the midgap band for GaAsN layers.

Figure 6a,b illustrate the energy band diagrams for 3- and 5-APJ at equilibrium without illumination. The band diagrams confirm proper band alignment at the contact semiconductor interfaces, ensuring efficient charge separation and transport. For the 3-APJ device, the band alignment is straightforward, facilitating effective carrier extraction. In contrast, the 5-APJ structure presents a more complex challenge due to the absence of tunneling junctions, which are typically employed in multijunction solar cells to mediate interlayer carrier transport. Without such junctions, optimal alignment requires precise tuning of carrier concentrations in each layer.

Device simulations prove critical in addressing this challenge, enabling the determination of effective carrier concentrations that enhance band alignment and device performance. Under illumination (Figure 6c,d), the band positions shift dynamically, promoting charge separation across the junctions. Notably, when the first junction is reverse-biased, the subsequent junction becomes forward-biased, enabling electrical transport as schematically presented in Figure 3. This behavior arises because the E_−_(k) band of the dilute nitride layer is electrically isolated from the contacts by the AlGaAs blocking layer.

Photon absorption in the HMA layers induces band shifts: for the junctions that are under reverse bias, the corresponding three energy bands moves downward (preserving the gaps at 0.63, 1.26 and 1.89 eV which correspond to a N content of 1.0%) injecting electrons into the E_−_(k) and E_+_(k) bands and holes into the E_−_(k) and E_v_(k). While for the junctions under forward bias, the three energy bands shift upward, injecting electrons into the E_−_(k) and E_+_(k) bands and holes into the E_−_(k) and E_v_(k). This creates an occupation inversion—electrons populate E_+_(k), holes occupy E_v_(k), and both carriers coexist in E_−_(k). Consequently, carrier tunneling occurs, bridging the reverse- and forward-biased junctions and enabling efficient carrier extraction. Lopez et al. have previously reported experimental demonstration of such tunnel current mechanism in a single p/n junction device [12]. This tunnel current mechanism is pivotal for the operation of these new types of devices, eliminating the need for tunnel junctions in conventional multijunction solar cells. By optimizing band alignment and increasing the number of stacked anti-parallel junctions, carrier generation and collection can be significantly improved. Furthermore, varying the nitrogen concentration across the junctions enables bandgap engineering, facilitating a simplified yet high-efficiency solar cell design.

### 3.3. Carrier Generation and Recombination Rates

Shockley-Read-Hall (SRH) recombination is a fundamental mechanism involving defect states within the bandgap or an additional energy band, as observed in our structure containing three optically active energy bands. This process governs electron-hole recombination through midgap states. SCAPS-1D facilitates the specification of SRH recombination parameters, including defect densities and energy levels, which are crucial for accurately modeling recombination processes in solar cells.

For HMAs layers, defect density varies depending on growth conditions and diluted nitrogen composition, typically ranging from 10^15^ to 10^18^ cm^−3^, encompassing nitrogen interstitials, Ga vacancies, and other defect complexes [36,38]. However, through optimized growth techniques such as molecular beam epitaxy (MBE) or metal–organic chemical vapor deposition (MOCVD), defect densities can be significantly reduced, reaching 10^14^ cm^−3^ [37].

**Figure 7:**
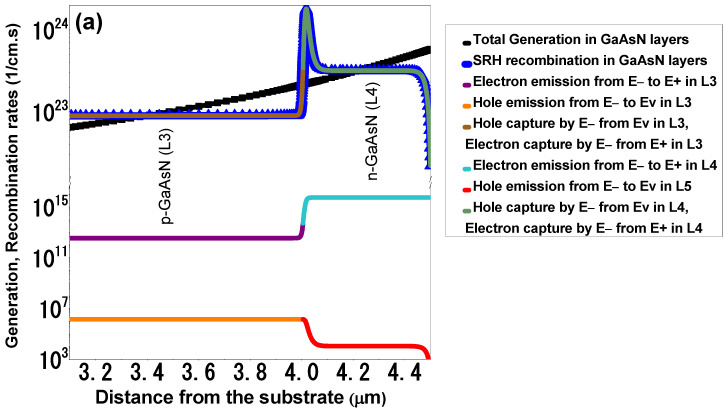

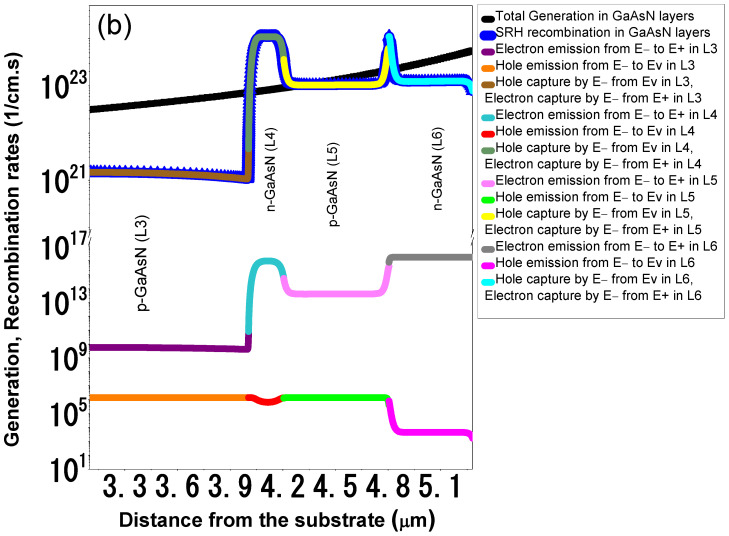
Generation and recombination rates analysis. (**a**) The response in the 3-APJ device. (**b**) The response in 5-APJ device.

We find that by introducing 1% nitrogen in GaAs, the E_−_(k) band is positioned at 1.26 eV above Ev(k) and 0.63 eV below the E_+_(k) band. This results in an optimum photon absorption via the E_−_(k) band, promoting efficient carrier generation while maintaining a stable and accurate growth process. Figure 7a,b present generation and recombination rate curves with illumination for the 3-APJ and 5-APJ devices, respectively, illustrating recombination rates within the HMAs with a modest defect density of 10^16^ cm^−3^.

The results presented in Figure 7a,b illustrate transitions across three energy bands. The emission and capture of electrons and holes between these bands are depicted in the figures, alongside the total recombination processes governed by the Shockley-Read-Hall mechanism in HMAs.

The total recombination rate in the 3-APJ of GaAsN layers structure is notably high in some regions of the bands. This increase can be attributed to several factors. (i) Introduction of the E_−_(k) band: The E_−_(k) band within the GaAsN energy gap introduces additional energy states, leading to more photogenerated carriers because of increased absorption. This increased density enhances the probability of recombination events. (ii) Thermal Effects: The presence of an additional energy band impacts the thermal properties of GaAsN. If the midgap band is fully occupied, it can act as a recombination center rather than facilitating efficient carrier transport, leading to increased thermal losses [39]. (iii) Defect Density: A high density of defect states (10^16^ cm^−3^), assumed in this calculation, also contributes to increased recombination rates.

In contrast, the 5-APJ structure exhibits lower recombination rates at the first and last GaAsN layers compared to the central p/n junctions. This behavior is attributed to blocking layers of AlGaAs positioned at the extremities. These blocking layers reduce recombination by spatially separating charge carriers, directing electrons and holes to different regions [8]. Furthermore, the blocking layers mitigate carrier trapping in localized states within HMAs, preserving carrier mobility and collection efficiency.

As observed in Figure 7b, recombination rates increase in layers not interfaced with a blocking layer, primarily due to elevated carrier density and thermal effects induced by the midgap band. Nonetheless, the 5-APJ device demonstrates higher efficiency than its 3-APJ counterpart, benefiting from enhanced total generation rates facilitated by additional GaAsN layers.

### 3.4. Record Comparison with III-V Multijunction Solar Cells

**Figure 8:**
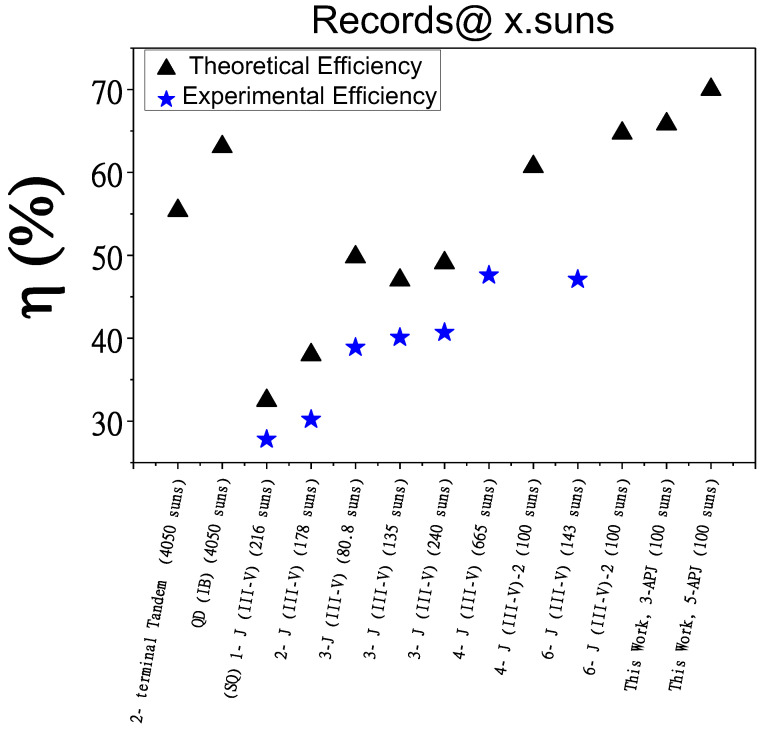
Performance comparison highlights the efficiency gains of our stacked anti-parallel junction’s design. Blue stars represent the experimental efficiency of multijunction solar cells. Black triangles are the theoretical calculation for each design. Theoretical efficiencies of the new stacked anti-parallel junctions solar cells in the present study under 100 suns are higher than experimental 6 tandem solar cells or theoretical single junction QD (IB) solar cells.

Finally, we compare the simulated performance of our multiband HMA-based APJ cells with previously reported record efficiencies of III-V multijunction solar cells. Figure 8 compares the efficiency of the APJ cells with various multijunction solar cell structures at different solar concentrations, highlighting the differences between traditional designs that use tunneling diodes to connect each junction and our innovative design of stacked multiple anti-parallel junctions free of tunneling diodes. This comparison includes both experimental and theoretical efficiency records. Table 3 provides a detailed overview of the illumination intensity and the corresponding references.

Starting with the Shockley-Queisser limit for a single GaAs p/n junction solar cell of 32.5% at 216 suns, the figure traces the progress in research aimed at increasing the number of p/n junctions and optimizing the selection of III-V materials. By adjusting the energy band gaps in different subcells, higher theoretical and experimental efficiencies were reported. Moreover, better cell performance with higher efficiencies can be achieved for multijunction solar cells with concentrated sunlight. Generally, increasing the number of junctions enhances solar cell efficiency. However, as mentioned earlier this approach is complex and costly. Our proposed structures demonstrate competitive theoretical efficiency at 100 suns. In particular, the tunneling junction-free 5-APJ structure can achieve a conversion efficiency of 70%. This structure consists of only six grown layers in total (adding back and front contacts), and three different materials, (GaAs, AlGaAs and GaAsN). Note that even the simplest conventional 3-junction structure achieving a theoretical efficiency of 50% requires at least 12 layers. In contrast, our 3-APJ solar cell only has 4 total grown layers and a theoretical efficiency of 65%. This detailed comparison underscores the advancements in concentration multijunction solar cell technology and the potential of our tunnel-junction-free design to set new efficiency records.

## 4. Conclusions

We propose a novel concept of a multiband solar cell structure formed by stacked anti-parallel junctions. The new device is designed by leveraging the unique electronic band structure of highly mismatched semiconductor alloys, which demonstrated a three active energy bands structure. Key to this design is a reverse-bias-induced tunnel current mediated by the three active energy bands, a phenomenon absent in p-n junctions in conventional (two energy bands) semiconductors. Using SCAPS-1D simulations, we optimized device parameters including layer thickness, doping level, and the number of stacked anti-parallel junctions, demonstrating that efficiency scales with the number of junctions. The efficiency obtained for an optimized structure with 5 anti-parallel junctions is 70% under 100-sun concentration.

Furthermore, this new solar cell architecture offers a potential fabrication advantage compared to high efficiency multijunction solar cells by reducing the number of distinct subcells and eliminating the need for complex tunnel junctions.

While the simulated efficiency is highly promising and surpasses many state-of-the-art theoretical and experimental benchmarks for multijunction structures, several critical challenges must be acknowledged. First, the performance is predicated on achieving nearly ideal material properties—such as defect-free interfaces, precise doping control, and the specific band alignment of the highly mismatched alloys—which are challenging to realize experimentally. Second, the scalability of the stacked anti-parallel junction design may be limited by series resistance accumulation and current-matching constraints in a real device, factors which are simplified in the 1D simulation environment.

Outlook for Real Device Fabrication: It is important to emphasize that the high PCE calculated in this study represents a theoretical potential under realistic conditions. The technological preparation of a real device will inevitably introduce limitations. Practical fabrication issues, including non-ideal ohmic contacts, interfacial recombination, material uniformity over large areas, and the thermal management required for concentrated sunlight operation will introduce losses not captured in our simulation. Future work must focus on the experimental realization of a single unit cell to validate the core tunneling mechanism and to quantify the performance gap between simulation and practice. Addressing these material growth and integration challenges will be essential for translating this innovative concept into a viable high-efficiency photovoltaic technology.

## Figures and Tables

**Table 1:** Optimized Parameters used in simulations for 3 and 5-APJ solar cells.

	Material	Carrier’s Concentration (cm^−3^)	Thickness (nm)
3-stacked anti-parallel junctions (3-APJ)	p-GaAs (L1)	$1\times 10^{19}$	3000
	n-AlGaAs (L2)	$1\times 10^{17}$	5.0
	p-GaAsN (L3)	$1\times 10^{18}$	1000
	n-GaAsN (L4)	$1\times 10^{17}$	500
	n-AlGaAs (L5)	$1\times 10^{19}$	5.0
5-stacked anti-parallel junctions (5-APJ)	p-GaAs (L1)	$1\times 10^{19}$	3000
	n-AlGaAs (L2)	$1\times 10^{18}$	5.0
	p-GaAsN (L3)	$1\times 10^{19}$	1000
	n-GaAsN (L4)	$1\times 10^{14}$	200
	p-GaAsN (L5)	$1\times 10^{17}$	600
	n-GaAsN (L6)	$1\times 10^{17}$	500
	n-AlGaAs (L7)	$1\times 10^{19}$	5.0
Total defect densities (cm^−3^)		$1\times 10^{16}$	
E_−_ level with respect to the top of E_v_ (eV)		1.26	
capture cross section electrons (cm^2^)		$1\times 10^{-15}$	
capture cross section holes (cm^2^)		$1\times 10^{-15}$	
Diluted nitrogen in GaAsN alloy (%)		1.0	
Al percent in AlGaAs blocking layer (%)		45	
Minority carrier’s lifetime (ns)		6.9	
Total defect concentration between interfaces (cm^−2^)		$1\times 10^{10}$	

**Table 2:** The photovoltaic parameters at different defect densities in the GaAsN layers.

Defect Density (cm^−3^)	V_oc_ (V)	$J_{\mathrm{sc}}\;\times 10^{3}$ (mA/cm^2^)	FF (%)	η (%)
$1\times 10^{14}$	1.72	5.27	87.33	79.27
$1\times 10^{15}$	1.64	5.26	86.64	75.17
$1\times 10^{16}$	1.56	5.16	86.54	70.02
$1\times 10^{17}$	1.45	4.61	82.66	55.69
$1\times 10^{18}$	1.31	3.73	78.11	38.32

**Table 3:** A Summary comparing ported experimental and theoretical efficiencies for the III-V MJSC and their illumination intensities and the 3- and 5-APJ cells in this study.

Cell Structure	Illumination (No. Suns)	Theoretical Cell Efficiency (%)	Experimental Cell Efficiency (%)	References
2-terminal Tandem	4050	55.4	--	[40]
QD IBSC	4050	63.1	--	[40]
(SQ) 1-J (III-V)	216	32.5	27.8	[41]
2-J (III-V)	178	38	30.2	[42]
3-J (III-V)	80.8	49.8	38.9	[13]
3-J (III-V)	135	47	40.1	[16]
3-J (III-V)	240	49.1	40.7	[16]
4-J (III-V)	665	--	47.6	[15]
4-J (III-V)-2	100	60.69	--	[43]
6-J (III-V)	143	--	47.1	[14]
6-J (III-V)-2	100	64.73	--	[43]
3-APJ	100	65.84	--	Present work
5-APJ	100	70	--	Present work

## Data Availability

The data supporting this project will be available at the request from the corresponding author due to privacy and confidentiality restrictions.

## Supplementary Materials

The following supporting information can be downloaded at: https://www.mdpi.com/article/10.3390/ma18245625/s1, Table S1: The possible transitions of carriers in the three active band gap materials; Table S2: Materials parameters considered to design the numerical model,. References [44,45] are cited in the supplementary materials.
