# Supplementary material for: Simulation of a Multiband Stacked Antiparallel Solar Cell with over 70% Efficiency
Source: Materials (Basel). 2025 Dec 15;18(24):5625. doi: 10.3390/ma18245625 (PMC12735105; doi:10.3390/ma18245625)
Supplement: Supplementary file 1 [file materials-18-05625-s001.zip › materials-4004627-supplementary.pdf]

Simulation of a Multiband Stacked Antiparallel Solar Cell with Over 70% Efficiency

Rehab Ramadan*[1,2], Kin Man Yu[3], and Nair López Martínez [1,4]

[1]Universidad Autónoma de Madrid, C/ Francisco Tomás y Valiente 7, 28048, Madrid, España

[2]Department of Physics, Faculty of Science, Minia University, Minia 61519, Egypt

[3]Department of Physics, National Sun Yat-sen University, Kaohsiung, Taiwan

[4] Departamento de Óptica y Fotónica de Superficies y Materiales, Instituto de Óptica - CSIC, C/ Serrano 121, 28006, Madrid, España.

*Corresponding author: rehab.ramadan@uam.es, rehabramadan@mu.edu.eg

Supplementary Materials: Simulation Methods

The numerical simulations in this work were performed using the Solar Cell Capacitance Simulator (SCAPS-1D), a well-established drift-diffusion solver that self-consistently solves the Poisson equation, continuity equations, and drift-diffusion equations to model solar cell operation under various conditions. This simulation tool enables the analysis of multi-layer device structures (up to seven layers) with customizable material parameters, including doping profiles, defect states, and band alignments. SCAPS-1D calculates key photovoltaic characteristics such as current-voltage (J-V) curves, capacitance-voltage (C-V) profiles, and quantum efficiency across temperatures ranging from 200 to 400 K.

The absorber material in our study is modeled as three-active-band semiconductors using the band anticrossing (BAC) framework, which describes the electronic structure modification induced by dilute nitrogen or oxygen incorporation in III-V or II-VI semiconductors. The BAC model predicts the formation of hybridized E+ and E- states through the interaction between the host conduction band and the impurity level, with their energy positions determined by the nitrogen/oxygen concentration. This three-band material enables additional sub-bandgap optical transitions beyond the conventional band-to-band absorption, specifically including E+→Ev, E+→E-, and E-→Ev transitions. To properly account for these multiple transition pathways, we implement Fermi-level splitting between the three bands and incorporate modeled data for absorption coefficients that capture the unique spectral response of these highly mismatched alloys.

The defect physics in these materials was carefully treated by specifying appropriate defect state densities and recombination mechanisms. The defect density (ρdef) was parameterized based on experimental characterization data, while recombination processes included both Shockley-Read-Hall and Auger mechanisms. The electrostatic potential distribution was determined by solving the Poisson equation with appropriate boundary conditions, where the charge contributions include free carriers (electrons and holes), ionized dopants, and defect states. Carrier transport was modeled through the continuity equations coupled with the drift-diffusion formalism, with generation rates calculated from the imported absorption spectra and recombination rates determined by the specified defect parameters.

The numerical implementation uses ohmic boundary conditions at the contacts and simulates standard AM1.5G illumination conditions (100 mW/cm²) for photovoltaic performance evaluation. Key material parameters, including the host bandgap, impurity concentration, and defect density, are taken from established theoretical and experimental studies to ensure physical relevance. This comprehensive simulation approach allows us to rigorously examine the photovoltaic properties of three-active-band solar cells while maintaining consistency with known material physics. The Poisson equation in SCAPS-1D describes the electrostatic potential (ϕ) distribution based on charge carrier densities (electrons n, holes p), doping profiles (N_D^+, N_A^-), and defect states (ρdef):

$$\frac{\partial^2 Q(x)}{\partial x^2} = -\frac{q}{\varepsilon_0 \varepsilon_r}\left[p(x) - n(x) + N_D^+(x) - N_A^-(x) + \frac{\rho_{def}}{q}\right] \qquad (S1)$$

In this context, Q represents the electrostatic potential, while ε_0 and ε_rr correspond to the vacuum permittivity and the semiconductor's relative permittivity, respectively. The electronic charge is denoted by q, with p and n signifying the hole and electron densities. The ionized donor and acceptor densities are given by N_D^+ and N_A^-, respectively. The defect charge density, ρ_{def}, plays a critical role in intermediate band solar cells (IBSCs), where intermediate band states contribute to ρ_def. The spatial coordinate x traces the device length, while t represents time.

SCAPS-1D numerically solves the coupled continuity equations for electrons and holes, incorporating both generation (G) and recombination (R) processes. For three-active-band solar cells, these equations must be extended to account for additional transitions involving the intermediate band (E-). Table S1 summarizes the possible transitions due to photon absorption in three-active band materials. Moreover, Figure S1 explains the three possible transitions and the splitting of the fermi level preserving distinct quasi-Fermi levels for each band.

Table S1. The possible transitions of carriers in the three active band gap materials

**possible transitions**	
**Optical Transitions**<br>These transitions describe how absorbed photons generate electron-hole pairs via the E-band:	**Thermal Transition s (Non-Radiative Processes)**<br>These transitions occur due to thermal energy or defect-assisted mechanisms:
**(a) Ev → E+ (Direct Band-to-Band Transition)**<br>**Mechanism**: High-energy photons (> bandgap *Eg*) excite electrons directly from Ev to E+.	**(a) E+ → E- (Thermal Relaxation or Trapping)**<br>**Mechanism**: Electrons in E+ relax to E- via phonon emission or defect trapping.

**(b) Ev band → E- band (First Sub-Bandgap Transition)**<br>**Mechanism**: Low-energy photons (Eg>hv>E₋-Ev) excite electrons from Ev to E-.	**(b) E- → Ev (Thermal Emission or Recombination)**<br>**Mechanism**: Electrons in E- recombine with holes in Ev (non-radiative or Shockley-Read-Hall (SRH) recombination).
(c) **E- → E+ (Second Sub-Bandgap Transition)**<br>**Mechanism**: Additional low-energy photons (Eg>hv>E-→E+) excite electrons from E- band to E+ band.	**(c) E+ → Ev (Direct or Trap-Assisted Recombination)**<br>**Mechanism**: Electrons in E+ recombine with holes in Ev (radiative or Auger recombination).

Summary of Continuity Equations for Three-Active-Energy Band Materials

(1) For electrons:

$$\frac{\partial n}{\partial t} = \frac{1}{q}\nabla \cdot J_n + G_{E_v\to E_+} + G_{E_-\to E_+} - R_{E_+\to E_v} - R_{E_+\to E_-} - R_{Auger} \quad \text{(S2)}$$

(2) For holes:

$$\frac{\partial p}{\partial t} = \frac{1}{q}\nabla \cdot J_p + G_{E_v\to E_+} + G_{E_v\to E_-} - R_{E_+\to E_v} - R_{E_-\to E_v} - R_{Auger} \quad \text{(S3)}$$

(3) For the third intermediate band:

$$\frac{\partial n_{IB}}{\partial t} = G_{E_v\to E_-} - R_{E_-\to E_v} - G_{E_-\to E_+} + R_{E_+\to E_-} \quad \text{(S4)}$$

The electron, hole current densities and electrons in the third intermediate band, J_n, J_p and n_{IB}, are modeled via the drift-diffusion formalism. Recombination rates for electrons and holes are defined as $R_{E_+\to E_v}$ and $R_{E_+\to E_-}$ for electrons and $R_{E_+\to E_v}$ and $R_{E_-\to E_v}$ for holes. While G denotes the generation rate, incorporating optical transitions (E_v → E_- and E_- → E_+). Moreover, the transition in the intermediate band is denoted by $G_{E_v\to E_-} - R_{E_-\to E_v}$ for the transition from *Ev* to *E-* and $G_{E_-\to E_+} + R_{E_+\to E_-}$ for the transition from *E-* to *E+*

The Lambert–Beer model was employed as the optical model. Illumination was applied from the front surface of the devices. Under these conditions, current-voltage curves in the dark and illumination states, as well as photovoltaic parameters (Voc, Jsc, FF, and η) were calculated at room temperature. Furthermore, the energy-band diagrams were built to illustrate the generation of carriers in the 3- and 5- stacked diodes devices.

Materials parameters used in this study were taken from various research articles and summarized in table S2.

Table S2. Materials parameters considered to design the numerical model

Parameter	GaAs substrate	AlGaAs	GaAsN
Band gap (eV)	1.42 [1-3]	1.98 [3, 4]	1.89 [5-8]
Electron affinity (eV)	4.07 [1-3]	3.57 [3, 4]	4.07 [5, 7]
Dielectric permittivity (relative)	13.1 [1, 3]	11.6 [3, 4]	12.4 [5]
CB effective density of state (cm^{-3})	4.70×10^{17} [1-3]	7.90×10^{17} [3]	4.66×10^{17} [5]
VB effective density of state (cm^{-3})	9.00×10^{18} [1-3]	3.30×10^{18} [3]	1.30×10^{19} [5]
Electron thermal velocity (cm/s)	4.40×10^{7} [1, 2]	3.45×10^{7} [3]	4.43×10^{7} [6]
Hole thermal velocity (cm/s)	1.80×10^{7} [1, 2]	0.58×10^{7} [3]	1.45×10^{7} [6]
Electron mobility (cm^2/V.s)	88.0×10^{2} [1-3]	206 [3, 4]	6.93×10^{3} [5, 6]
Hole mobility (cm^2/V.s)	400 [1-3]	96.4 [3, 4]	3.98×10^{2} [5, 6]

References

[1] C.F. Kamdem, A.T. Ngoupo, F.K. Konan, H.J.T. Nkuissi, B. Hartiti, J.-M. Ndjaka, Indian Journal of Science and Technology, 12 (2019) 37.

[2] M. Tridane, A. Malaoui, S. Belaaouad, (2022).

[3] M. Levinshtein, Handbook series on semiconductor parameters, World Scientific, 1997.

[4] S. Adachi, Properties of aluminium gallium arsenide, IET, 1993.

[5] M.S. Shur, Handbook Series on Semiconductor Parameters, Vol. 2: Ternary and Quaternary III-V Compounds, World Scientific, 1996.

[6] J. Thordson, O. Zsebök, U. Södervall, T. Andersson, Materials Research Society Internet Journal of Nitride Semiconductor Research, 2 (1997) e8.

[7] R. Chtourou, F. Bousbih, S.B. Bouzid, F. Charfi, J. Harmand, G. Ungaro, L. Largeau, Applied physics letters, 80 (2002) 2075-2077.

[8] N. López, L. Reichertz, K. Yu, K. Campman, W. Walukiewicz, Physical Review Letters, 106 (2011) 028701.
